# *Pseudostellariawuyishanensis*, a new species of Caryophyllaceae from Fujian, China

**DOI:** 10.3897/phytokeys.181.67436

**Published:** 2021-08-25

**Authors:** Xiao Luo, Qi-Yi Yang*, Zhe Zhang, Pan Zhu, Liang Ma, Xin-Yan Chen, Shu-Yi Lin, Shi-Pin Chen

**Affiliations:** 1 Fujian Provincial Forestry Survey and Design Institute, Fuzhou 350002, China; 2 College of Forestry, Fujian Agriculture and Forestry University, Fuzhou 350002, China; 3 College of Forestry, Southwest Forestry University, Kunming 650224, China; 4 College of Horticulture and Landscape Architecture, Southwest University, Chongqing 400700, China; 5 Key Laboratory of National Forestry and Grassland Administration for Orchid Conservation and Utilization at College of Landscape Architecture, Fujian Agriculture and Forestry University, Fuzhou 350002, China; 6 Sanming City Garden Center, Sanmin 365000, China; 7 Middlebury College, Vermont 05753, USA

**Keywords:** Caryophyllaceae, Fujian, Pseudostellaria, Wuyishan National Park

## Abstract

*Pseudostellariawuyishanensis*, a new species from the Wuyishan National Park, Fujian, China, is described and illustrated. Morphologically, *Pseudostellariawuyishanensis* resembles *P.heterantha*. However, the new species can be distinguished by presence of stolons, 1 line of hairs on the stem, smaller leaf blades, shorter pedicels, and ovary with 2 styles.

## Introduction

*Pesudostellaria* Pax is a small genus that belongs to the tribe Alsineae in Caryophyllaceae (Bittrich 1993; Tang et al. 1996). This genus can be easily distinguished from other genera in Caryophyllaceae from the presence of the flesh root tuber. In addition, the vast majority of species in the genus have cleistogamous flower and chasmogamous flowers that have petals with two sections (Zeng et al. 2016). Some recent molecular studies show that this group is non-monophyletic which includes a new described genus *Hartmaniella* and 2 species *Stellariaamericana* (Porter & B.L.Rob.) Standl. and *Arenariaprzewalskii* Maxim. nested within *Pseudostellaria* (Greenberg and Donoghue 2011; Zhang et al. 2017). Russian botanist Turczaninow (1842) first used *Krascheninikovia* Turcz. ex Fenzl for this genus, but this name was in fact a previous synonym for *Eurotia* Adans and did not comply with the international nomenclature regulations. *Pseudostellaria* was established by Pax in 1934 as the new name, which has been used until now (Schischkin and Komarov 1936; Ohwi 1937; Mizushima 1965). Currently, the genus is represented by ca.22 accepted species that are widely distributed all over the world, with 20 species in eastern and northern Asia, 1 species in Europe, and 1 species in North America. (Zeng et al. 2016; Zhang et al. 2017).

Since the turn of the 21^st^ century, 3 new species of *Pesudostellaria* have been established in China. Jin and Ding (2003) described *P.zhejiangensis* X.F Jin & B.Y Ding from the Zhejiang province based on its decumbent creeping stems, obtuse petals, and compressed seeds with a narrow wing. Lian (2009) described *P.polymorpha* W. Z. Di & Y. Ren based on the regular variation in its floral morphology from stem apex to base. Xia et al. (2011) described *P.tianmushansis* Xia et al. based on its several tubers in a row, obovate with a bi-lobed apex petal and tubercles awned seeds. 12 species of *Pesudostellaria* have been recorded in China out of which 5 species are endemic.

During an investigation of wild plants in Fujian Province, southeastern China, that took place in May 2019 and October 2020, an unknown species of *Pseudostellaria* was collected from the deciduous broad-leaved forest in Wuyishan National Park. We found that it resembles *P.heterantha Pax* but has stolons, 1 line of hears in the stem, smaller leaves, and shorter pedicels. Therefore, we established it as a new species.

## Material and methods

All general morphological data were obtained by observation of specimens during fieldworks and AU, FJIDC, IBSC, KUN, LE herbaria. Terminologies used in the present study follows the *Flora of China* (Lu and Rabeler 2001) and additional consultation of online databases, including Chinese Field Herbarium and Plant Photo Bank of China.

## Taxonomy

### Pseudostellaria wuyishanensis

Taxon classificationPlantaeCaryophyllalesCaryophyllaceae

X. Luo & Q.Y. Yang sp. nov.

B466D9C6-9170-5644-A4EA-A9363E172BBF

urn:lsid:ipni.org:names:77219367-1

Figs 1 , 2

#### Type.

China. Fujian: Wuyishan National Park, on rocks along a stream, ca.1700 m a.s.l, 1 May 2019, Xiao Luo et Qiyi Yang20190501 (***holotype***: FAFU!; ***isotype***: FAFU!)

**Figure 1.**
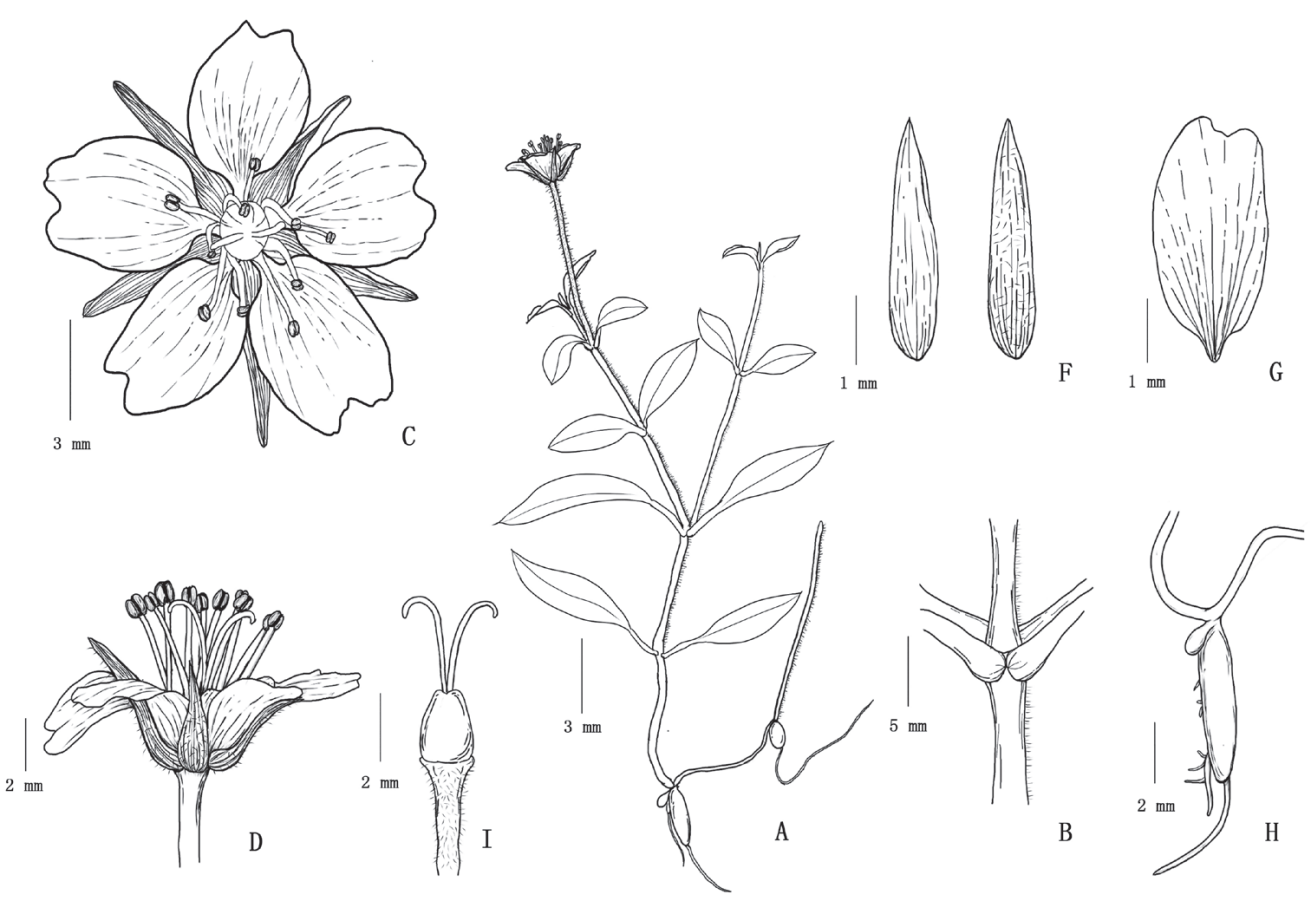
*Pseudostellariawuyishanensis* sp. nov. **A** plant **B** stem with one line of hair **C** flower **D** flower in side view **E** calyx **F** petal **G** tuber **H** gynoecium of chasmogamous flower.

**Figure 2.**
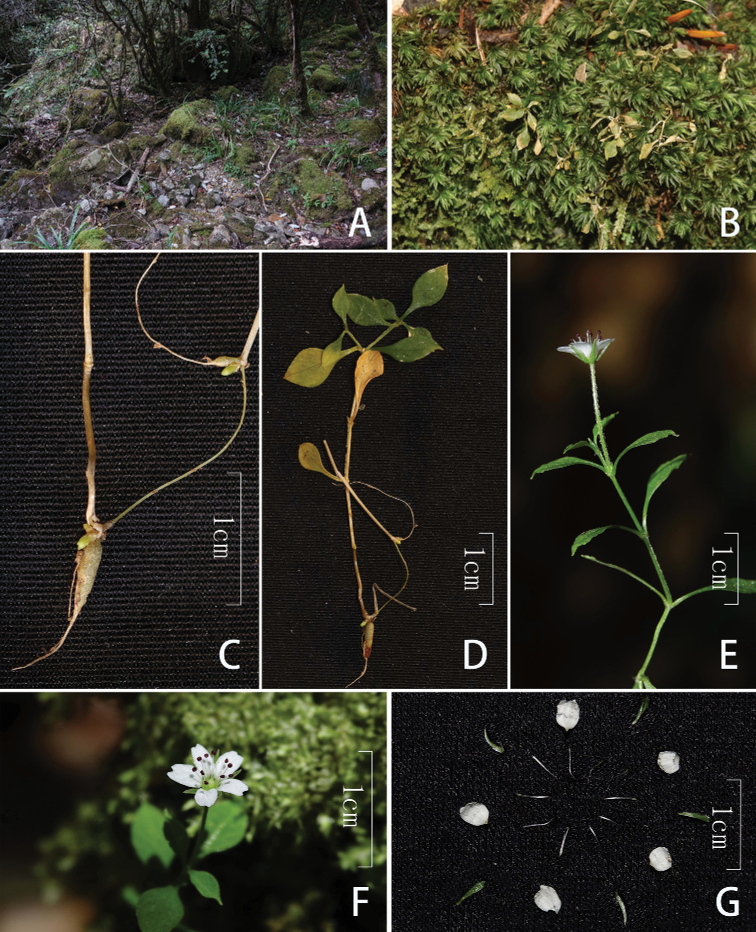
*Pseudostellariawuyishanensis* X. Luo, Q.Y. Yang **A** habitat **B** habit **C** tuber **D** plant **E** flowering plant **F** flower **G** all parts of flower.

#### Diagnosis.

*Pseudostellariawuyishanensis* can be distinguished from *P.heterantha* by several morphological features and distribution (Table 1). *P.wuyishanensis* has stolons (vs. no stolon in *P.heterantha*), 1–1.6 × 0.5–0.7 cm (vs. 2–2.5 × 0.8–1.2 cm in *P.heterantha*) leaf blade, ca. 2 cm long (vs. 3–3.5 cm long in *P.heteranth*) pedicel and is 6–7 cm tall with 1 line of hairs (vs. 8–15 cm tall with 2 lines of hairs in *P.heterantha*). *P.wuyishanensis* only distribute in Wuyishan National Park, Southeastern China (vs. Northern and Southwestern China in *P.heterantha*)

**Table 1.** Morphological comparison of *Pseudostellariawuyishanensis*, *P.heterantha*.

Characters	* P. wuyishanensis *	* P. heterantha *
**Stem**	has stolons,branched at apex, 6–7 cm tall,with 1 line of hairs	no stolon, branched at base, 8–15 cm tall, with 2 lines of hairs
**Leaf blade**	1–1.6 × 0.5–0.7 cm	2–2.5 × 0.8–1.2 cm
**Pedicel**	ca.2 cm long	3–3.5 cm long
**Sepal**	abaxially pilose, margin glabrous	abaxially pilose, margin ciliate
**Ovary**	2 styles	2 or 3 styles
**Distribution**	Fujian(Southeastern China)	Northern and Southwestern China

#### Description.

Plants perennial. Root tubers green, fusiform, 0.4–0.6 × 0.2–0.3 cm. Stem erect, 6–7 cm tall, slender, unbranched at base, apex false dichotomous branched, stoloniferous, with 1 line of hairs. Leaves opposite, entire, 1–1.6 × 0.5–0.7 cm; proximal middle leaves oblanceolate, base attenuate into a petiole, apex acute; distal leaves ovate, shortly petiolate, membranous, both surfaces glabrous, the adaxial green, the abaxial viridescent, apex acute, usually with mucro ca.0.5 mm, sparsely ciliate at base, pinnately veined, lateral veins 3–4 pairs, inconspicuous. Chasmogamic flowers terminal or axillary, solitary; pedicel erect, ca. 2 cm long, pilose; sepals 5, green, lanceolate, ca. 3 mm, abaxially slightly pilose, margin membranous, glabrous; petal 5, oblong, slightly longer than sepals, ca. 4 mm, apically emarginate, base with a short claw; stamens 10, shorter than petals, ca. 4 mm; filament glabrous; anthers purple-red, reniform; ovary coniform, ca. 2 × 0.9 mm, with 2 thin styles to 3 mm, revolute, longer than the ovary, ovules numerous. Cleistogamous flowers and fruits not seen.

#### Distribution and habitat.

The new species is endemic to the Wuyishan National Park, Fujian Province. The plant grows in the deciduous broad-leaved forest at 2000 m in elevation. The dominant species of the community include *Pinustaiwanensis* Hayata (Pinaceae), *Lithocarpusharlandii* (Hance) Rehder (Fagaceae), Buxussinicavar.parvifolia M. Cheng (Buxaceae), *Veratrumschindleri* Loes (Melanthiaceae), and *Dichocarpumfranchetii* (Finet&Gagnepain) W.T. Wang & Hsiao (Ranunculaceae).

#### Phenology.

Flowers were observed in June.

#### Conservation status.

There is only one known location and fewer than 50 individuals of *P.wuyishanensis* found during our fieldworks in the Wuyishan National Park in both 2019 and 2020. But the investigation has not been through enough to fully understand the species natural distribution. According to IUCN Red List criteria (2012), this new species should be assessed as Data Deficient (DD; criteria B1ab(i–v) + 2ab(i–v)).

#### Etymology.

The specific epithet ‘*wuyishanensis*’ refers to Wuyishan National Park, the locality of the type collection.

## Discussion

The new species morphologically resembles *P.heterantha* in the leaf shape, terminal chasmogamous flowers with pilose pedicel, and white emarginate petal. The two taxa differ in that the stem of *P.wuyishanensis* is shorter, conspicuously stoloniferous, apex false dichotomous branched, and only has 1 line of hairs, while that of *P.heterantha* is longer, solitary, branched at base, and has 2 line of hairs; the leaf blades of *P.wuyishanensis* is smaller and the pedicel is shorter.(Table 1).

Ohwi (1937) regarded *P.maximowicziana* (Franch. & Sav.) Pax and *P.himalaica* (Franchet) Pax as the synonym of *P.heterantha*. The view was also approved by Mizushima (1965) and Lu (1998). However, some research results published in recent years do not support such a view (Chen et al. 2014; Zeng et al. 2016; Zhang et al. 2017). Zeng et al. (2016) suggested taking *P.maximowicziana*, *P.himalaica*, and *P.heterantha* as independent species respectively. None of the 3 species were collected in Fujian province or the surrounding area.

**Figure 3.**
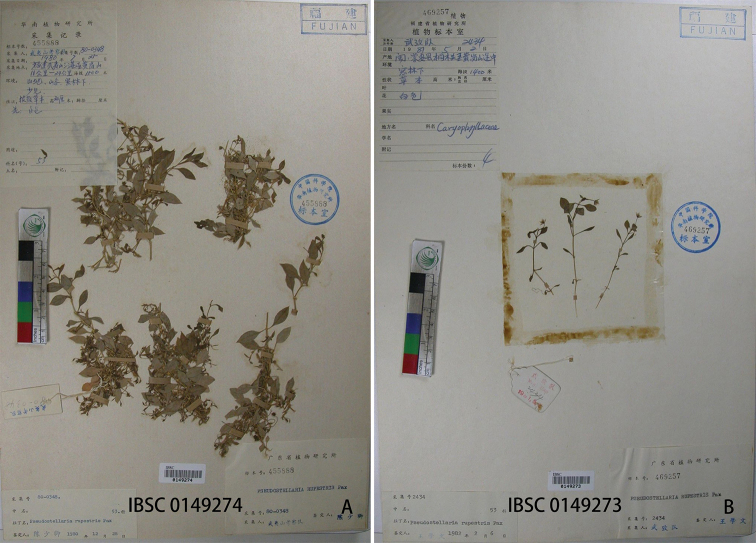
Specimens of *Pseudostellariawuyishanensis* collected in 1980s.

We only found two *Pseudostellaria* sp. specimens, IBSC 0149273 and IBSC 0149274 (Fig. 3) were collected in the Wuyishan National Park. Former researchers have identified them as *P.rupestris* (Turczaninow) Pax or *P.heterophylla* (Zeng et al. 2016). Morphologically, we found that the arrangement, shape and hairs of the leaves of these specimens were completely different from those two species, and the morphology of each part was consistent with *P.wuyishanensis*. In addition, the distribution location of *P.rupestris* was far away from the collection site. Considering all the factors, we believe that these specimens are in fact *P.wuyishanensis*.

### Key to the Chinese species of *Pseudostellaria*

**Table:** 

1	Seeds with persistent anchor-shaped barb	*** Pseudostellaria rupestris ***
–	Seeds with awned tubercles	**2**
2	Stems with apical 2 pairs of leaves larger, approximate, decussate	*** P. heterophylla ***
–	Stems not as above	**3**
3	Chasmogamic flowers with petals apex 2-lobed	**4**
–	Chasmogamic flowers with petals apex entire, sometimes emarginate	**7**
4	Root tubers several in a row	**5**
–	Root tubers solitary	**6**
5	All leaves linear or lanceolate-linear, sessile	*** P. sylvatica ***
–	All leaves narrow elliptic-lanceolate, with short petiole	*** P. tianmushanensis ***
6	Chasmogamic flowers with sepals 4, petals 4,stamens 8	*** P. helanshanensis ***
–	Chasmogamic flowers with sepals 5, petals 5, stamens 10	*** P. japonica ***
7	Chasmogamic flowers with sepals glabrous	*** P. tibetica ***
–	Chasmogamic flowers with sepals abaxially pubescent	**8**
8	Stem repent	**9**
–	Stem erect	**10**
9	Leaves pubescent in both side; seeds flat, with narrow wings	*** P. zhejiangensis ***
–	Leaves ciliate; seeds reniform or subglobose	*** P. davidii ***
10	Stem pubescent; Leaves both surfaces pubescent	*** P. himalaica ***
–	Stems with 1 or 2 line of hairs; base of the leaves sparsely ciliate	**11**
11	Stem has stolons, with 1 line of hairs; pedicel short, ca. 2 cm	***P.wuyishanensis* (sp. nov.)**
–	Stem has 2 line of hairs; pedicel longer than 3 cm	**12**
12	Petals spatulate or obovate	*** P. maximowicziana ***
–	Petals oblong-oblanceolate	*** P. heterantha ***

## Supplementary Material

XML Treatment for Pseudostellaria wuyishanensis
